# Efgartigimod for patients with thymoma associated generalized myasthenia gravis during the perioperative period: a four-case report

**DOI:** 10.3389/fimmu.2025.1627584

**Published:** 2025-10-02

**Authors:** Liuli Ren, Ling Wei, Song Jiang, Hongru Li, Anguo Chen, Juanjuan Zhang

**Affiliations:** ^1^ Department of Pharmacy, First Affiliated Hospital of Anhui Medical University, Hefei, China; ^2^ Department of Neurology, First Affiliated Hospital of Anhui Medical University, Hefei, China; ^3^ Department of General Thoracic Surgery, First Affiliated Hospital of Anhui Medical University, Hefei, China

**Keywords:** efgartigimod, thymoma, myasthenia gravis, perioperative period, case report

## Abstract

**Background:**

Thymectomy is one of the main treatments for thymoma associated myasthenia gravis, but there is a risk of acute exacerbation of myasthenia gravis symptoms during the perioperative period of thymoma. Therefore, perioperative management is very important. We reported four cases of thymoma associated generalized myasthenia gravis who were treated with efgartigimod before and after the perioperative period. The myasthenic symptoms of the patients were quickly controlled before surgery, and the patients successfully completed the surgery without experiencing myasthenic crisis or respiratory failure after surgery.

**Case report:**

Four patients diagnosed with thymoma associated generalized myasthenia gravis were all acetylcholine receptor(AChR) antibody-positive, with case 2 also positive for Titin and anti-RyR antibodies. Prior to surgery, all patients exhibited varying degrees of myasthenic symptoms, with case 2 experiencing a myasthenic crisis induced by glucocorticoid pulse therapy before the operation. Admitted to our multidisciplinary treatment group for myasthenia gravis, all four patients received 1 to 2 treatments with efgartigimod before thymoma surgery, which led to an improvement in myasthenia symptoms and a significant reduction in MG-ADL and QMG scores. All four patients underwent video-assisted thoracoscopic mediastinal tumor resection under general anesthesia, followed by the 3rd to 4th treatments with efgartigimod postoperatively, with no perioperative myasthenic crisis occurring in any of the patients. Apart from case 1, who developed a new pulmonary infection postoperatively and experienced a temporary fluctuation in myasthenia symptoms, the other three patients had no severe complications. Follow-up visits at one month and three months postoperatively showed that three of the patients achieved minimum symptom expression (MG-ADL score ≤1).

**Discussion:**

Efgartigimod provides new insights into the perioperative management of thymomas in patients with thymoma associated generalized myasthenia gravis.

## 1 Introduction

Myasthenia gravis (MG) is an autoimmune disease characterized by acquired neuromuscular junction transmission disorders mediated by autoantibodies (1). All skeletal muscles of the body can be affected, with clinical symptoms mainly manifesting initially as diplopia and ptosis of the upper eyelid, gradually progressing to weakness of the limbs, trunk, and respiratory muscles. Some patients may start with systemic symptoms, and in severe cases, it can be life-threatening (1, 2). About 80% of MG patients have thymic abnormalities, including thymic hyperplasia and thymoma, among which patients with MG associated with thymoma account for about 10% to 15% of MG patients (3, 4). Thymectomy is the standard treatment for thymoma associated myasthenia gravis, which can improve long-term prognosis by removing the source of autoantibody production and abnormal lymphoid tissue (5, 6). However, perioperative stress, the use of anesthetic drugs, and infection are factors that can easily trigger acute exacerbation of myasthenic symptoms. The incidence of postoperative myasthenic crisis can reach 7%-33%, significantly increasing the need for mechanical ventilation and hospital mortality (7–9). Current perioperative management largely relies on plasma exchange (PE), intravenous immunoglobulin (IVIG), and pulse therapy with glucocorticoids (10), but the above-mentioned regimens have limitations such as delayed onset of action, increased risk of infection, and hemodynamic instability, necessitating the exploration of safer and faster new immune-modulating strategies.

Efgartigimod is the world’s first approved neonatal Fc receptor (FcRn) antagonist, which accelerates the catabolism of pathogenic IgG antibodies (including AChR antibodies) by blocking the FcRn-IgG complex recycling pathway (11). In the global multicenter, randomized, double-blind, placebo-controlled Phase III clinical trial (ADAPT) and its open-label extension study (ADAPT+), efgartigimod demonstrated significant efficacy as a monotherapy. Compared to the placebo group, the efgartigimod group showed sustained and clinically meaningful improvements in MG-ADL, QMG, and MG-QOL15r scores (12, 13). Currently, evidence on the application of efgartigimod in the perioperative management of thymoma-associated myasthenia gravis (TAMG) remains limited to small samples or case reports. Wang’s group used an innovative “2 + 2 regimen” efgartigimod which resulted in rapid, significant, and safe improvement of neuromuscular symptoms during the perioperative period, substantially reducing the risk of postoperative myasthenic crisis (14, 15). However, a prior study reported two cases of acute symptom exacerbation in TAMG patients following efgartigimod treatment previously (16). Therefore, more clinical studies are necessary to further substantiate the use of efgartigimod in the perioperative management of thymoma-associated gMG. Leveraging real-world data, this retrospective case series analyzed four thymectomy-treated gMG patients managed with a flexible efgartigimod regimen and extended follow-up. The study evaluated the protocol’s impact on perioperative symptom control, postoperative complications, medication safety, and longer-term outcomes, providing clinical insights to optimize perioperative management in thymoma-associated gMG.

## 2 Case presentation

### 2.1 Case 1 A

49-year-old male patient presented with right ptosis of unknown cause three months ago, accompanied by weakness in the upper limbs and neck muscles, showing diurnal fluctuation and worse in the evening. Symptoms progressively worsened, with subsequent left ptosis. The patient visited a local hospital where a neostigmine test was positive, and serum antibody testing for MG revealed positive AChR antibodies (8.02nmol/L). His chest CT identified a mediastinal mass. Therefore he was admitted into our multidisciplinary treatment group for thymoma surgery. Based on comprehensive evaluations, the diagnosis was thymoma-associated gMG, with Myasthenia Gravis Foundation of America IIIb (MGFA IIIb). The patient had no history of thyroid disease, rheumatologic disorders, or other comorbidities and had not received corticosteroids or immunosuppressants preoperatively. Preoperative assessments showed a MG-ADL score of 4 and a QMG score of 13. Due to the risk of myasthenic crisis during thymoma surgery, the neurology department administered efgartigimod (10 mg/kg weekly×2) preoperatively. After two doses, his ptosis and limb weakness were improved (MG-ADL score 2, QMG score 6). The patient underwent thoracoscopic mediastinal tumor resection under general anesthesia in the department of thoracic surgery and the pathology confirmed type AB thymoma. The drainage tube was removed on postoperative day 3 without complications. A third dose of efgartigimod was administered on postoperative day 4. At one week postoperatively, the MG-ADL score was 1 and the QMG score was 6, and the patient was discharged. Two weeks post-discharge, the patient was readmitted to neurology department for a fourth efgartigimod dose but developed a pulmonary infection with worsened MG symptoms with a MG-ADL score of 4 and a QMG score of 14. Efgartigimod was withheld. Aggressive antibiotic therapy was used and resulted in improvement of his pulmonary infection. Post-discharge medications included pyridostigmine 75 mg every 6 hours, prednisone 25mg once daily, with an increase of 5mg per week until reaching the target dose, and tacrolimus 1mg twice daily. Follow-up after surgery showed a MG-ADL score of 3 one month postoperatively and a MG-ADL score of 1 three months postoperatively.

### 2.2 Case 2 A

61-year-old male patient presented with accompanied by left eyelid ptosis, blurred vision, and difficulty in swallowing without any apparent cause. The symptoms worsened 10 days prior to admission, accompanied by chest tightness. The patient was treated at a local hospital where a neostigmine test was positive. The electromyography indicated postsynaptic membrane disorder. Serological tests for MG showed positive anti-AChR antibodies (>20nmol/L), positive Titin antibodies, and positive anti-RyR antibodies. A CT scan of the chest revealed a mass in the anterior mediastinum on the left side and changes indicative of bilateral pulmonary inflammation. The local hospital administered pyridostigmine and high-dose steroid pulse therapy. The patient was admitted to our multidisciplinary treatment group for thymoma surgery. Based on the examination results from the local hospital, the diagnosis of thymoma-associated gMG was confirmed, with MGFA V. The patient had a history of hypertension and cerebral infarction, no thyroid disease, and no rheumatological tests were performed. Upon admission, the MG-ADL score was 8, and the QMG score was 14. Treatment included pyridostigmine 60mg every 8 hours and methylprednisolone 500mg for pulse therapy, which was gradually tapered. On the fourth day of steroid pulse therapy, the patient experienced a myasthenic crisis characterized by respiratory distress, a decrease in oxygen saturation to 88%, and blood gas analysis showing an oxygen partial pressure of 49.2mmHg and a carbon dioxide partial pressure of 48.14mmHg. The patient was treated with a non-invasive ventilator and transferred to the neurology ICU for treatment, where efgartigimod (10mg/kg) was administered intravenously once a week. After two doses of efgartigimod, the patient’s clinical symptoms significantly improved, with a MG-ADL score of 1 and a QMG score of 5. After 3 days of treatment with efgartigimod, the patient underwent thoracoscopic mediastinal tumor resection under general anesthesia and the pathology confirmed type A thymoma. The drainage tube was removed on the second postoperative day without any perioperative complications. The third dose of efgartigimod was administered on the fourth postoperative day, and one week after surgery, the MG-ADL score was 1 and the QMG score was 5. The patient had a concurrent pulmonary infection before surgery, which did not worsen during the course of the disease, and no other infections occurred in other parts of the body. He was discharged on postoperative day 6, and he was prescribed pyridostigmine 60mg every 8 hours and prednisone 25mg once daily, with an increase of 5mg per week until reaching the target dose. Follow-up after surgery showed a MG-ADL score of 1 one month postoperatively and a MG-ADL score of 0 three months postoperatively.

### 2.3 Case 3 A

74-year-old male patient presented with ptosis of the upper eyelid and weak chewing, accompanied by weakness in both lower limbs and labored breathing half a month ago. Symptoms worsened with activity and improved with rest, exhibiting a characteristic pattern of being milder in the morning and worsening in the evening. Upon admission, relevant examinations were conducted, including a positive neostigmine test and electromyography indicating a postsynaptic membrane lesion at the neuromuscular junction. Low-frequency repetitive nerve stimulation of both facial nerves and both accessory nerves showed a significant decrement phenomenon. Serum antibody testing for MG revealed a positive AchR antibody (14.50nmol/L). A CT scan of the chest suggested an anterior mediastinal mass, which was considered likely to be a thymoma. Based on the examination results, the patient was diagnosed with thymoma-associated gMG, classified as MGFA IIIb. The patient had no history of thyroid disease, rheumatic disease, or other underlying conditions. Prior to surgery, the patient had not taken corticosteroids or other immunosuppressive drugs. The MG-ADL score was 5, and the QMG score was 17. Considering the risk of myasthenic crisis associated with thymoma surgery, the patient was administered efgartigimod (10mg/kg qw×2) in the neurology department preoperatively. After one dose of efgartigimod, the patient’s ptosis and limb weakness symptoms improved compared to before, with an MG-ADL score of 4 and a QMG score of 9. The patient was then transferred to the thoracic surgery department and underwent thoracoscopic mediastinal tumor resection under general anesthesia after two doses of efgartigimod, and the pathology confirmed type AB thymoma. The drainage tube was removed one day after surgery, with no perioperative complications. Efgartigimod was administered for the third time on the fifth day postoperatively. One week after surgery, the MG-ADL score was 0, and the QMG score was 10, with no significant infections observed during the course of the disease. The patient was discharged on postoperative day 14 with a prescription for oral pyridostigmine bromide 60mg every 8 hours and prednisone 20mg once daily, with an increment of 5mg per week until reaching the target dose. Follow-up after discharge showed a MG-ADL score of 0 one month postoperatively and a MG-ADL score of 0 three months postoperatively.

### 2.4 Case 4 A

50-year-old female patient presented with left ptosis without any apparent cause half a month ago, characterized by improvement in the morning and worsening in the evening. She had normal limb activity but was prone to fatigue and occasionally experienced chest tightness that worsened with activity. The patient was admitted to the neurology department. Relevant examinations upon admission indicated a positive neostigmine test, electromyography suggesting a postsynaptic membrane lesion at the neuromuscular junction, and serum antibody testing revealed positive anti-AChR antibodies (17.40nmol/L). A CT scan of the chest suggested an occupation in the right anterior mediastinum, likely a thymoma. Based on the examination results, the patient was diagnosed with thymoma-associated gMG, classified as MGFA IIa. The patient had no rheumatic diseases or other underlying conditions, with elevated only TSH levels. She had not taken corticosteroids or other immunosuppressive drugs before surgery and was assessed with a MG-ADL score of 4 and a QMG score of 11. Considering the risk of myasthenic crisis associated with thymoma surgery, efgartigimod (10mg/kg qw×1) was administered in the neurology department preoperatively. After one dose of efgartigimod, the patient’s ptosis and limb weakness improved, with a MG-ADL score of 2 and a QMG score of 7. The patient was then transferred to the thoracic surgery department and underwent thoracoscopic mediastinal tumor resection under general anesthesia. The pathological diagnosis of the resected specimen was a type B1 thymoma. The drainage tube was removed two day after surgery, without any perioperative complications. The second dose of efgartigimod was administered on the fifth postoperative day, with a MG-ADL score of 0 and a QMG score of 7 one week postoperatively. The patient was discharged on postoperative day 6 and was readmitted on postoperative day 12 for administration of the third dose of efgartigimod. Two weeks postoperatively, the MG-ADL score remained 0 alongside a QMG score of 6. There were no significant infections observed during the disease. After discharge, the patient was prescribed oral pyridostigmine bromide 60mg every 8 hours and prednisone 20mg once daily, with an increase of 5mg per week until reaching the target dose. Follow-up after surgery showed a MG-ADL score of 0 one month postoperatively and a MG-ADL score of 3 three months postoperatively.

**Figure 1:**
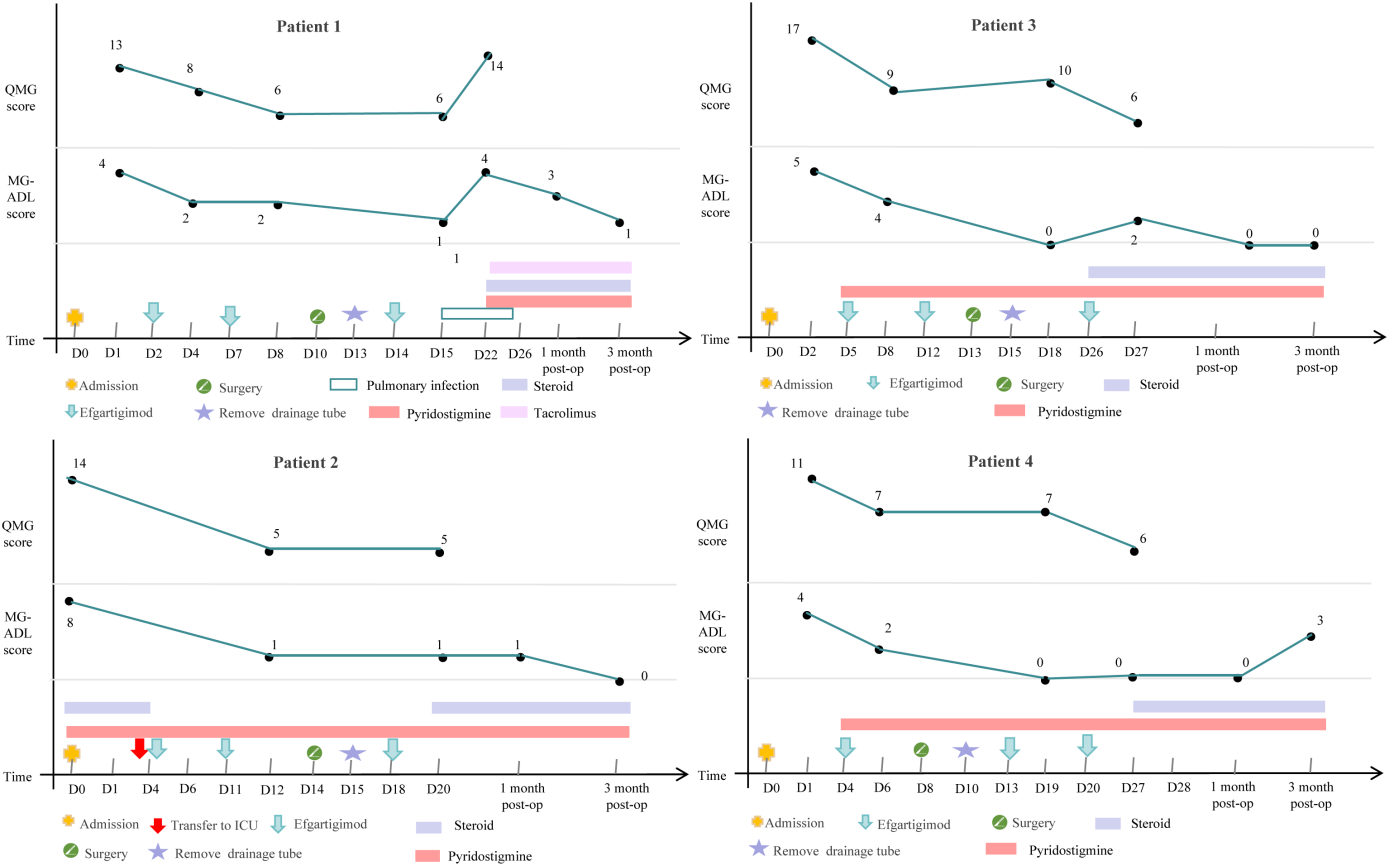
Schematic illustration of the changes in treatment process and MG-ADL and QMG scores.

The demographic and clinical characteristics of 4 patients are shown in **Table 1**, the changes in treatment process and MG-ADL and QMG scores during the disease are shown in **Figure 1**.

**Table 1:** Demographic and clinical characteristics of 4 patients.

Case	Case 1	Case 2	Case 3	Case 4
Age,years	49	61	74	50
Sex	Male	Male	Male	Female
Height,cm	178.5	172	172	160
Weight,kg	96	80	65	80
AChR-Abs	Positive	Positive	Positive	Positive
Other-Abs	N	Positive Titin Ab and anti RyR Ab	N	N
MGFA class at screening	IIIb	V	IIIb	IIa
Duration of MG, month (m)	3	1	0.5	0.5
Other underlying diseases	Hypertension	Hypertension, Cerebral infarction, Pulmonary infection	N	N
MG therapy preoperative				
Pyridostigmine bromide	N	Y	Y	Y
Steroid	N	Y	N	N
Any NSIST	N	N	N	N
Efgartigimod dosage during perioperative period				
Preoperative(doses)	2	2	2	1
Postoperative(doses)	1	1	2	2
Pathological types of thymoma	AB	A	AB	B1
Postoperative complications	Pulmonary infection	N	N	N
Other adverse drug events	N	N	N	N

AChR-Abs, acetylcholine receptor antibodies; Other-Abs, other-antibodies; MGFA, Myasthenia Gravis Foundation of America; MG, myasthenia gravis; NSIST, non-steroidal immunosuppressant therapy; N, no; Y, yes. Efgartigimod of one dose means 10mg/kg qw*1.

## 3 Discussion

In patients with gMG, the co-occurrence of thymoma is relatively common and is associated with more severe disease and a relatively poor prognosis (3, 4). Thymectomy is an important treatment option for these patients (1), however, the surgery itself carries certain risks. Particularly in patients with MG, surgery may trigger or exacerbate myasthenic crisis, increasing the risk of perioperative complications (7–9). IVIG and PE serve as conventional perioperative treatment options, with demonstrated efficacy in rapidly alleviating symptoms and reducing the incidence of myasthenic crises. However, the clinical application of IVIG faces accessibility challenges: its production relies on plasma from healthy donors, which has limited availability, and the manufacturing process is complex and costly. Furthermore, IVIG has broad indications, besides MG, it is also commonly used for other neuroimmune disorders such as Guillain-Barré syndrome and chronic inflammatory demyelinating polyneuropathy, as well as hematological diseases, further straining its supply. Plasma exchange requires specialized equipment, trained personnel, and specific replacement fluids, and carries risks such as hypocalcemia, hypotension, and secondary infections, which also limit its applicability. As a novel therapeutic agent, efgartigimod works by competitively binding with high affinity to the neonatal FcRn, blocking the recycling of IgG and achieving “endogenous clearance” of IgG, thereby reducing pathogenic antibody levels and improving myasthenic symptoms. It has been hailed as “plasma exchange in a vial”. In the ADAPT and ADAPT+ studies, efgartigimod has shown tolerability and efficacy in gMG patients (12, 13). Currently, it is widely used in patients with acetylcholine receptor (AChR)-antibody-positive gMG. However, its application in TAMG remains primarily based on small-scale studies and case reports (**Table 2**).

**Table 2:** Literature comparison of studies on efgartigimod for thymoma-associated myasthenia gravis.

Study	Kawama et al., 2023 (16)	Wang et al., 2024 (14)	Wang et al., 2025 (15)	Ren et al., 2025 (this study)
Study Type	case report	case report	prospective,multicenter, phase II clinical trial	case report
Number of Patients	2	1	40	4
Gender(F/M)	1/1	1/0	23/17	1/3
Age range(years)	52-53	47	20-70	49-74
Antibody Type	AChR-Abs(+)	AChR-Abs(+), RyR-Abs(+)	AChR-Abs(+)	AChR-Abs(+), One case with AChR/Titin/RyR-Abs (+)
Clinical stage	Non-perioperation	Perioperation	Perioperation	Perioperation
Dosage regimen	Cycle regimen(10mg/kg qw, interval of 6–7 weeks)	Preoperative:2 Postoperative:2	Preoperative:2 Postoperative:2	Preoperative:1–2 Postoperative:1-2
Outcomes	QMG, Anti-AChR-antibody	MG-ADL, ADR	MG-ADL, QMG, MGC, IgG/AChR-Ab, POMC, Postoperative hospital stay duration, ADR	MG-ADL, QMG, Time for removing drainage, POMC, Postoperative hospital stay duration, ADR
Follow up period	Not reported	Not reported	2 months	3 months
Novelty	First to report a potential safety signal: clinical exacerbation related to antibody overshoot	The first reported case	Establishes efficacy and safety of a fixed 4-cycle regimen in the perioperative setting	1.First report of efficacy in a patient with a complex antibody profile 2. Provides real-world evidence for a flexible, individualized dosing strategy 3.Offers longer-term (3-month) follow-up data

AChR-Abs, acetylcholine receptor antibodies; QMG, QuantitativeMyasthenia Gravis; MG-ADL, Myasthenia Gravis Activities of Daily Living; MGC, the Myasthenia Gravis Composite scale; ADR, adverse reaction; POMC, postoperative myasthenic crisis.

This case series reports on four patients with thymoma-associated gMG who were treated with efgartigimod perioperatively, all of whom were positive for AChR antibodies. Case 2 was also positive for Titin and RyR antibodies and experienced a myasthenic crisis induced by glucocorticoid pulse therapy before surgery. We administered efgartigimod treatment one or two doses to the four patients before thymoma surgery. The average MG-ADL score decreased from 5.25 ± 1.89 at admission to 2.25 ± 1.26, and the average QMG score decreased from 13.75 ± 2.60 at admission to 6.75 ± 1.71. After significant improvement in muscle weakness symptoms, the patients underwent thoracoscopic mediastinal tumor resection under general anesthesia and received their third to fourth doses of efgartigimod postoperatively. None of the four patients experienced a myasthenic crisis or respiratory failure after surgery. One week postoperatively, the average MG-ADL score was 0.50 ± 0.58, and the average MG-ADL score was 7.00 ± 2.16. Except for case 1, who developed a new pulmonary infection postoperatively and experienced a transient fluctuation in myasthenic symptoms, the other three patients showed significant improvement in myasthenic symptoms without any serious drug-related adverse events. All four patients had their drainage tubes removed within three days after thymectomy, and none experienced a myasthenic crisis, the postoperative length of stay was 8.00 ± 4.00 days. It is worth noting that Case 2, who was transferred to the ICU preoperatively due to a myasthenic crisis, showed rapid improvement in myasthenic symptoms after two doses of efgartigimod treatment. The patient successfully underwent thymectomy and achieved a favorable postoperative recovery. Notably, the observed risks of postoperative myasthenic complications (0% vs. reported rates of 7~33%), ICU admission rates (0% vs. 24.6%), and the postoperative hospital stay (8.00 ± 4.00 days vs. 9.8 ± 5.9 days) in our case series are favorably lower than the previous studies, particularly those undergoing surgery following a recent crisis (7–9, 17). All four patients reported a positive experience throughout the course of treatment. This was consistently reflected in the notes regarding their rapid symptomatic improvement, expressed satisfaction with the speed of recovery, and regained ability to perform daily activities, which significantly contributed to their preoperative confidence and postoperative morale.

A Phase II clinical study confirmed that the perioperative “2 + 2” dosing regimen of efgartigimod (two doses before and two doses after surgery) significantly reduced the incidence of postoperative myasthenic crisis (POMC) to 10% and achieved a postoperative remission rate of 82.5% in patients with TAMG, with a favorable safety profile (15). Previously, their team reported another case on the use of efgartigimod for treating TAMG (14). In our reported 4 cases of patients with thymoma-associated gMG, the application of efgartigimod significantly and rapidly controlled the patients’ symptoms, providing an opportunity for thymoma surgery. A flexible dosing strategy was adopted, in which the number of preoperative doses was tailored according to the individual’s clinical response to efgartigimod, while postoperative dosing was adjusted based on whether myasthenic symptoms worsened or complications such as infection emerged after surgery. This strategy represents a departure from the standard “2 + 2” dosing protocol, providing enhanced flexibility and the capacity for a more tailored treatment regimen. Significantly, Case 2, who was positive for anti-AChR, Titin, and anti-RyR antibodies and poorly responsive to steroid therapy, experienced a myasthenic crisis prior to surgery. The presence of multiple antibodies often suggests more severe disease, a higher risk of myasthenic crisis, and complicates both treatment and prognosis. However, treatment with efgartigimod led to rapid symptomatic improvement, which allowed the surgery to proceed successfully and resulted in a stable condition postoperatively. Di Stefano et al. reported three patients with both AChR-seropositive gMG and anti-GAD-seropositive stiff-person syndrome who were treated with efgartigimod, and all patients showed improvement in symptoms of both disorders after two cycles of treatment (18). Additionally, efgartigimod has also shown benefits in therapy-refractory autoimmune encephalitis with coexistent NMDAR and LGI1 antibodies, as well as in Morvan syndrome with positive VGKC, LGI1, and CASPR2 antibodies (19, 20). By reducing all IgG antibodies, efgartigimod offers a promising strategy for treating complex, multi-antibody-positive autoimmune disorders beyond classic gMG, as highlighted by these findings.

This study is limited by its retrospective nature and the associated incomplete data collection. Most notably, post-treatment AChR antibody titers were not routinely available, as they were not part of standard clinical care. The relationship between anti-AChR antibody titers and disease severity of MG remains controversial (21). Interestingly, Kawama et al. reported two cases where clinical deterioration following efgartigimod was accompanied by an increase in anti-AChR antibody titers beyond pre-treatment levels (16). A similar paradoxical rise in antibodies has been documented after plasma exchange (22), suggesting a complex immunological rebound phenomenon. This paradoxical phenomenon underscores the necessity for further investigations into antibody titers following efgartigimod treatment in patients with thymoma-associated gMG. Moreover, it focused solely on four cases with a relatively short follow-up period. Indeed, this preliminary data on efgartigimod in thymoma associated gMG should be confirmed on large cohorts of patients in randomized and controlled studies.

In conclusion, efgartigimod shows potential in the perioperative treatment of thymoma-associated gMG patients undergoing thymectomy, and the risk of postoperative infection should be noted. Future research should focus on determining the optimal timing, dosage, and combination strategies with other treatment methods to further optimize the treatment plan for such patients.

## Data Availability

The original contributions presented in the study are included in the article/Supplementary Material. Further inquiries can be directed to the corresponding author.
